# An Investigation into the Use of Stimulant Therapy during Pregnancy

**DOI:** 10.1155/2012/308952

**Published:** 2012-09-06

**Authors:** Natalie Shields, Rexford Muza, Christopher Kosky, Adrian J. Williams

**Affiliations:** ^1^School of Medicine, King's College London, London WC2R 2LS, UK; ^2^Sleep Disorders Centre, St Thomas' Hospital, London SE1 7EH, UK

## Abstract

*Introduction*. A lack of documentation of stimulant use during pregnancy means that doctors have difficulty advising narcoleptic and hypersomnolent patients. *Objectives*. To investigate the use of stimulant therapy in narcoleptic and hypersomnolent patients during pregnancy. *Method*. A search of clinic letters at a tertiary sleep clinic identified women who became pregnant whilst receiving stimulant therapy between 01/09/1999 and 18/11/2010. Fifteen patients were included in a telephone survey. *Results*. There were 20 pregnancies. The reported advice received with regards to stimulant use was variable. In 7 pregnancies, medication was stopped preconceptually: 1 had a cleft palate and an extra digit 6 had good foetal outcomes. In 8 pregnancies, medication was stopped postconceptually: 1 had autism and attention-deficit hyperactivity disorder; 7 had good foetal outcomes. In 5 pregnancies, medication was continued throughout pregnancy: 2 ended in miscarriage; 1 was ectopic; 2 had good foetal outcomes. The most common symptom experienced was debilitating hypersomnolence. *Conclusion*. There are no standardised guidelines for use of stimulants during pregnancy. Women have significant symptoms during pregnancy for which there is an unmet clinical need. More research is needed into whether medication can be safely continued during pregnancy, and if not, when it should be discontinued. Better standardized advice should be made available.

## 1. Introduction

In practice, the advice given to women with narcolepsy and idiopathic hypersomnolence is to discontinue stimulant therapy during pregnancy because of fear of potential teratogenicity. Some patients chose to remain on stimulant therapy during their pregnancy because of intolerable sleepiness. There is a lack of documentation on how women cope with their symptoms during pregnancy, and in practice, doctors find it difficult to advise these patients. At the tertiary sleep clinic used in this study, there are approximately 110 patients currently being followed up for narcolepsy and idiopathic hypersomnolence. 42 of those patients are females and of fertile age.

Narcolepsy is a cause of excessive daytime sleepiness (EDS) distinguished by abnormal intrusions of rapid eye movement (REM) sleep such as cataplexy, sleep paralysis, and hypnagogic hallucinations during wakefulness [1]. Idiopathic hypersomnolence is also a cause of EDS but patients do not meet the diagnostic criteria for narcolepsy. There is sleep inertia; EDS, and naps are usually more prolonged than in narcolepsy and less refreshing [2]. 

The American Sleep Disorders Association Standards of Practice (1994) [3] states that the aim of therapy is to alleviate EDS, thereby allowing the fullest possible return of normal function for patients. Amphetamine-like drugs, such as dextroamphetamine, methamphetamine, methylphenidate, and the wake-promoting compound modafinil, are the most commonly prescribed medication for EDS [4]. Mazindol is a less commonly used stimulant. Newer antidepressant medications, such as venlafaxine, are also effective agents useful for treatment of the other features of narcolepsy sleep paralysis and hallucinations [5]. Behavioural approaches include: good sleep hygiene, patient education, coping strategies, employment that protects a regular sleep-wake schedule, and support groups [5].

The U.S. Food and Drug Administration's categories of drugs indicate the potential for teratogenicity a drug has [6]. The 2007 American Association of Sleep Medicine review of treatment for narcolepsy categorizes amphetamine, methylphenidate, modafinil, fluoxetine, and venlafaxine as category C which states that “Animal reproduction studies have shown an adverse effect on the foetus and there are no adequate and well-controlled studies in humans, but potential benefits may warrant use of the drug in pregnant women despite potential risks.” Therefore, these drugs are contraindicated in pregnancy [7]. The National Toxicological Program Centre for the Evaluation of Risks to Human Reproduction [8, 9] concluded that although there are a number of studies investigating the effects of the use of amphetamines and methylphenidate during pregnancy, the data was largely insufficient to support clear conclusions, primarily because of inadequate study designs. Similarly, The National Teratology Information Service [10] reported that the limited data available to assess the safety of methylphenidate in pregnancy do not suggest a significantly increased risk of adverse foetal outcome but are too limited to exclude any increase in risk. It is not known if modafinil crosses the placenta, but it is considered to be of low risk in pregnancy [11]. Pemoline is thought to be the safest stimulant during pregnancy but due to the potential for hepatic toxicity, it is no longer available in some countries [12].

The morbidity of sleepiness and the mother's risk of suffering an accident as a result of sleepiness should be weighed against the foetus' possible risk of problems as a result of exposure to intrauterine stimulants [3]. If a patient should decide to come off all stimulant therapy during their pregnancy and whilst breastfeeding, the following instructions were suggested by the Sleep Disorders Center of Louisiana State University [13] (Table 1).

**Objectives**

To investigate the following:

- advice given to patients who use stimulant therapy with regards to falling pregnant;
- timing of patients' discontinuation of medication;
- symptoms that patients experience during their pregnancy and their management of these symptoms;
- outcome of the pregnancies and the health of the infants;
- opinions the patients hold about the use of stimulants during pregnancy.

## 2. Method

A search of the clinic letters from a tertiary sleep centre in London was conducted using the word “pregnant” as the search criteria between 01/09/99 and 18/11/10. Ethical clearance was granted before the commencement of data collection. The search returned 164 letters which were reviewed alongside the patients' medical record. Fifteen patients were identified as having had a pregnancy whilst using stimulant therapy; they were included in the study. The others were excluded because they either had a pregnancy but were not on stimulant therapy or there was no pregnancy.

For each of the fifteen patients, medical records were used to obtain information regarding diagnosis, stimulant therapy regimes, previous medical history, and other medications. They were then telephoned and asked a series of questions (Appendix) pertaining to the objectives of the study. Verbal consent was obtained from each patient.

## 3. Results 

Fifteen subjects were telephoned: one had 5 pregnancies, another had 2 babies, and 13 had one baby each whilst under the care at the Sleep Disorders Unit. A total of 20 pregnancies were included.

### 3.1. Diagnosis, Medication, and Medical History

Table 2 shows the diagnosis of each patient, including their mean sleep latency test (MSLT) results and number of naps where sleep onset REM (SOREM) occurred. The stimulant therapy, dose, and timing of its discontinuation are shown as well as their other medical conditions, medications, caffeine use, and smoking status.

In summary, 4 participants had narcolepsy, 6 had narcolepsy with cataplexy, and 5 had idiopathic hypersomnolence. The medications taken were modafinil (6/15), dexamphetamine (3/15), methylphenidate (2/15), mazindol (1/15), venlafaxine and modafinil (1/15), and modafinil and citalopram (1/15), modafinil changed to methylphenidate (1/15). Although antidepressants venlafaxine and citalopram are not stimulants, the women taking these were also taking modafinil, and so they have been included as they may have important effects. 7 women used caffeine during their pregnancy, 4 did not, and for 4 it is unknown if they used caffeine. 7 women did not smoke at the time of the study, and for 7 the smoking status was unknown.

### 3.2. The Advice Given to Patients Regarding Stimulant Therapy and Pregnancy

Table 3 shows the varying advice given to the patients regarding stimulant therapy and pregnancy. Out of the 20 pregnancies included in the study, only 4 were planned with the sleep centre physicians. Of the 16 pregnancies not planned with the sleep center, advice came from other sources including General Practitioners and Obstetricians.

At this sleep center advice is also given when stimulants are prescribed for the first time. Women of a child-bearing age are advised to stop stimulants if they become pregnant. Some patients may not have received this additional advice if they had started stimulant therapy at other centers. Clinic letters indicate that patients who fell pregnant were discharged until they had finished breastfeeding and could recommence medication. This action resulted in many women feeling concerned about discontinuing stimulant therapy and the withdrawal of clinic support with no alternative options offered.

### 3.3. Timing of Discontinuation of Medication

The ranges of number of weeks before and after conception that medication was discontinued for each pregnancy are shown in Table 4.

### 3.4. Symptoms That Patients Experience and Their Management of These Symptoms

All the women reported that they had to nap during the day. The naps varied from taking a scheduled nap during a lunch hour to feeling they “slept throughout their entire pregnancy.” Some women had to stop working whilst others were able to continue up to the thirty-eighth week. The ability to remain at work depended largely on support from their employer and colleagues. Two women reported drinking caffeine. Most of the women who drove, stopped. Another woman reported avoiding sedentary activities (Table 5).

### 3.5. Outcome of the Pregnancies and Health of the Infants

Table 6 shows how many infants were healthy and how many had “nontrivial” health issues. Trivial health issues included eczema and lactose intolerance.

The mother (F) of the infant born with a cleft palate and an extra digit stopped taking modafinil, 100 mg daily, 4 weeks prior to conception. She reported drinking caffeine during her pregnancy. The mother (B) of the infant born with autism and attention deficit hyperactivity disorder stopped taking dexamphetamine, 10 mg BD, after conception, she was unable to recall how many weeks pregnant she was. The same mother had another baby with no health issues; and she stopped taking dexamphetamine prior to conception but could not recall how many weeks before. One mother (D) remained on mazindol throughout her pregnancy; this baby was healthy but born at thirty four weeks and admitted to Special Care Baby Unit. One woman (A) experienced two miscarriages and one ectopic pregnancy whilst continuing to take dexamphetamine she also had two healthy infants where she stopped taking dexamphetamine at five and eight weeks of gestation. One mother (N) remained on methylphenidate throughout her pregnancy; interestingly the baby was born at thirty nine weeks with a healthy weight.

Of the seventeen babies born to the fifteen women, sixteen were of normal birth weights, and one was 4lb 2oz (1.81 kg) born at thirty four weeks. The length of gestation was within normal ranges except the baby born at thirty four weeks. This baby was delivered early due to preeclampsia. The mode of delivery was natural in seven cases. The others were all delivered by Cesarean section. One mother elected for Cesarean section because of worries over cataplectic attacks during labour. The other Cesarean sections were for reasons reportedly unrelated to narcolepsy.

### 3.6. Opinions about the Use of Stimulants during Pregnancy the Patients Hold

Eleven women reported that they would stop their medication during pregnancy. Three women said they would not have another baby because of intolerable symptoms during pregnancy.

## 4. Discussion

### 4.1. The Advice Given and When Medication Was Discontinued

This study highlights that in practice women are given varying advice from doctors and that many pregnancies are not planned with the sleep and obstetric physicians. This can cause confusion and concern for the women and indeed for some health care professionals. There is a need for standardised advice for stimulant therapy use and pregnancy. Advice on how to reduce risk of narcoleptic-related accidents during pregnancy and with a new born baby, such as that given by the Louisiana State University [13], would be useful.

### 4.2. Symptoms Experienced

Narcoleptic women have significant symptoms during their pregnancy which impact their quality of life, for which there is an unmet clinical need for safe treatment. EDS was the most common symptom, and for some, it was so disabling that they had to leave employment.

The attitude of employers was an important factor affecting the women's ability to remain at work. Employer education about the symptoms and the need for scheduled naps may help the patient remain at work [14].

Patient support groups can be effective for many medical conditions; some women may benefit from having a forum where coping strategies can be discussed and support given.

### 4.3. The Outcome of Pregnancies and the Health of Infants

Some significant and worrying medical conditions were developed by the infants born to the patients in this sample. A chi-squared test was carried out on the results which, due to the small sample size, did not find a significant difference between the group where medications were stopped prior to conception and the groups where medication was either stopped after conception or continued throughout pregnancy. Due to a small sample size and the nature of this retrospective study where important variables could not be controlled, no connections between the stimulant used and the timing of discontinuation and the infants health can be made. However, it does highlight an important point that these drugs could potentially cause these medical conditions, and more research is needed into their teratogenicity. 

In the UK, adverse events relating to medicines are reported using the Yellow Card Scheme provided by the Medicines and Healthcare products Regulatory Agency. The case of the infant with a cleft palate and extra digit was reported. The child with autism and ADHD was unfortunately not reported at the time (a decade ago). 

### 4.4. Limitations of the Study

This is a retrospective study which, due to recall bias, means that some information is lacking. For example, it would be interesting to know if the women experienced spontaneous conceptions or if there were any problems conceiving. A family history of pregnancy complications or foetal abnormalities is lacking as the obstetric records could not be obtained.

Given the retrospective and uncontrolled nature of this study, the search used to identify women who had had pregnancies whilst on stimulant therapy might not have included women who may have had miscarriages between visits to the clinic. Additionally, other women may not have been to the clinic during pregnancy or may have themselves stopped medications prior to conception and not had any problems with their pregnancy. If this was the case for some women, this would not be mentioned in medical letters and so not captured by the search method. 

The use of telephone conversations, as opposed to written questionnaires, had both benefits and drawbacks. Because of the sensitive nature of the information and the small sample size, it was deemed more personal to speak to each person in order to get a better response rate. As there is little research into the management of narcolepsy during pregnancy, it was difficult to predict the experiences each woman may have had, and therefore, the line of questioning needed to be flexible to gain an understanding of what was important to each individual. For these reasons, it was beneficial to have open questions that were more conversational rather than targeted.

Because it is not a controlled study, there are variables such as the stimulant medication, dose of medication, point at which each mother stopped taking the medication, and age, weight, and smoking status of the mother that must be taken into account. 

## 5. Conclusion

There is little research into and no standardized guidelines for the pharmacological treatment of narcolepsy during pregnancy. The study shows that women have significant symptoms that can affect their quality of life during pregnancy for which there is an unmet clinical need and better support is needed. The opinions of the women were united that they did not want to risk the health of their baby during a hypothetical future pregnancy. The advice given to the women with regards to stopping their medication was variable. This is due to a lack of evidence of the effect of stimulant therapy on foetal development. More research is needed into whether medication can be safely continued during pregnancy and if not, when it should be discontinued. Better standardized advice should be made available to patients. Referral to patient support groups could be useful as the patients might feel isolated by a lack of awareness and support.

## Figures and Tables

**Table 1:** Instructions for pregnant narcoleptic women [13].

	Advice for safety whilst off stimulant medication:
	(i) Make arrangements to have others drive for you
	(ii) Take showers instead of baths to reduce the risk of drowning
	(iii) No swimming for the time being
	(iv) Avoid heights or other situations where having a sleep attack or cataplectic attack could be hazardous
	(v) Take naps during the day to reduce the likelihood of sleep attacks
	Advice regarding the handling of your baby:
	(i) Wash the baby on a mat with a bowl of water so that should you have a sleep attack or cataplectic attack the baby will come to no harm
	(ii) Do not wash the baby in the bath
	(iii) Breastfeed in a lying position to reduce the risk of injury to the baby should you have a sleep attack or cataplectic attack
	(iv) Use a wheel baby carriage to transport the baby around the house to help reduce the risk

**Table 2:** Diagnosis, mean sleep latency test (MSLT) results, number of sleep onset REM (SOREM), stimulant therapy, dose, timing of discontinuation of stimulant, medical history, other medications, caffeine use, and smoking status of each patient.

	Diagnosis	MSLT score	SOREM	Medication	Dose	Timing of discontinuation of stimulants	Other medical conditions	Medication other than stimulant	Caffeine use	Smoking status
A	Narcolepsy	Diagnosis made prior to 2000 MSLT result not available		Dexamphetamine	10 mg BD	2004 1st baby: discontinued 8/40 2009 2nd baby: discontinued 5/40 Continued medication with 2 miscarriages and 1 ectopic	Nil	Nil	Caffeine not used	Smoking status not recorded
B	Idiopathic hypersomnia	4 min	0/4 naps	Dexamphetamine	10 mg BD	Postconceptually but not clear what gestation	Asthma	Seretide, salbutamol, aminophylline montelukast	Caffeine not used	Nonsmoker
C	Idiopathic hypersomnia	2, 4 min	1/5 naps	Modafinil	100 mg BD	Discontinued at 12/40	Obstructive sleep apnoea, Gestational Diabetes	CPAP, metformin	Caffeine use unknown	Nonsmoker
D	Narcolepsy with Cataplexy	Diagnosis made prior to 2000 and referral to this sleep centre		Mazindol	2 mg BD	Continued throughout pregnancy	Hypertension	Fluoxetine, omeprazole, atenolol	Caffeine used	Smoking status not recorded
E	Idiopathic hypersomnia	3, 8 min	1/5 naps	Modafinil	400 mg OD	Stopped modafinil when fell pregnant; started methylphenidate at 17/40	Nil	Nil	Caffeine not used	Smoking status not recorded
F	Hypersomnia	11 min	0/4 naps	Modafinil	100 mgs OD	4 weeks preconception	Costochondritis, asthma	Salbutamol	Caffeine used	Nonsmoker
H	Narcolepsy	Diagnosis make by a private neurologist		Modafinil	200 mg BD	Discontinued 2/40	Eczema	Nil	Caffeine used	Nonsmoker
I	Narcolepsy with Cataplexy	0, 6 min	3/4 naps	Modafinil citalopram	200 mg BD 20 mgs daily, respectively	Discontinued preconceptually	Previous glandular fever	Nil	Caffeine used	Smoking status not recorded
J	Narcolepsy with Cataplexy	3, 6 min	3/4 naps	Modafinil	100 mg BD	Discontinued at 6/40	Nil	Nil	Caffeine not used	Smoking status not recorded
K	Likely narcolepsy	2, 7 min	1/5 naps	Modafinil	300 mg OD	Discontinued 3 weeks preconception	Nil	Nil	Caffeine use unknown	Ex-smoker
L	Narcolepsy with Cataplexy	2, 3 min	3/4 naps	Methylphenidate	10 mgs BD	Discontinued 6 months perceptually	Nil	Nil	Caffeine used	Nonsmoker
M	Narcolepsy with cataplexy	4, 9 min	3/4 naps	Dexamphetamine	5 mgs BD	Discontinued 7/40	Simple solar lentigos on upper back	Nil	Caffeine used	Smoking status not recorded
N	Narcolepsy with cataplexy	Diagnosis made by paediatric neurologist: notes unavailable		Methylphenidate	36 mg OD	Continued throughout pregnancy	Nil	Nil	Caffeine use unknown	Smoking status not recorded
O	Narcolepsy	1, 6 min	3/4 naps	Modafinil and venlafaxine	200 mgs daily	Discontinued 4 weeks preconceptually	Polycystic ovaries, eczema	nil	Caffeine not used	Nonsmoker
P	Narcolepsy	0 h 7 m 0 s	2/4 naps	Modafinil	100 mgs BD	Discontinued 2 weeks preconceptually	Nil	nil	Caffeine use unknown	Nonsmoker

**Table 3:** Advice given to patients.

Advice given	Number of women
Discontinue medication 3 months prior to conception	3
Discontinue medication once pregnant	3
Reduce medication slowly once pregnant	2
Stay on medication; baby may have withdrawal symptoms	1
No advice	6

**Table 4:** Timing of discontinuation of stimulants.

Medication stopped before or after conception	Range of number of weeks	Number of pregnancies
Preconception	2 weeks–6 months	7
Postconception	2–12 weeks	7
Continued throughout pregnancy		6

**Table 5:** Symptoms experienced during pregnancy.

Symptoms during pregnancy	Number of women
EDS	14
EDS during 1st trimester only	2
Symptoms abated	1

**Table 6:** Pregnancy outcome.

Pre-conception discontinuation of medication		Post-conception discontinuation of medication		Medication continued throughout pregnancy	
7		7		6	

Healthy	Unhealthy	Healthy	Unhealthy	Healthy	Unhealthy

6	1	6	1	3	3

3 modafinil	1 modafinil	3 Dexamphetamine	1 Dexamphetamine	1 maxindol born 34 weeks	2 dexamphetamine miscarriage
	Cleft palatte & extra digit on foot		Autism & ADHD		
1 Modafinil & Citralopram		3 modafinil			
				1 methylphenidate	1 dexamphetamine ectopic
1 Venlafaxin & modafinil				1 modafinil (discontinued at 3 weeks) then methylphenidate started at 17 weeks	
1 methlyphenydate					

## Appendix

### Telephone Survey

I am going to ask you some questions about your child's birth:

when was your baby born?
how much did the baby weigh?
how was the birth? Caesarean? naturally?
how many weeks were you when the baby was born?
were there any health issues with your baby when they were born?

Now I have some questions about your sleep disorder and medication before you were pregnant:

before your pregnancy, do you remember what medication you were taking?
how were your symptoms?
how did you manage? naps? driving? caffeine?

Now I have some questions about your sleep disorder during the pregnancy and postnatally:

what advise were you given about becoming pregnant and taking stimulant medication?
when did you stop taking your medication?
number of weeks: before conception? after conception? not at all?
when did you restart your medication?
how did you feel during your pregnancy? cataplexy? sleepiness?
how did you manage your symptoms? naps? driving? working? caffeine?

Could I now ask some questions about your opinion of stimulant medication during pregnancy?

If you were to plan another child whilst you were on stimulant medication would you stop your medication or continue? When would you stop your medication?
